# Case report: Acute ST-elevation myocardial infarction and cardiogenic shock caused by a giant right sinus of Valsalva aneurysm and right coronary artery compression

**DOI:** 10.3389/fcvm.2022.1013044

**Published:** 2022-10-18

**Authors:** Lianyue Ma, Jianmin Yang, Yan Liu, Fang Wang, Tongtao Liu, Ying Wang, Hourong Sun, Cheng Zhang, Yun Zhang

**Affiliations:** ^1^The Key Laboratory of Cardiovascular Remodelling and Function Research, Chinese Ministry of Education, Chinese National Health Commission and Chinese Academy of Medical Sciences, The State and Shandong Province Joint Key Laboratory of Translational Cardiovascular Medicine, Department of Cardiology, Qilu Hospital of Shandong University, Jinan, China; ^2^Department of Radiology, Qilu Hospital of Shandong University, Jinan, China; ^3^Department of Cardiac Surgery, Qilu Hospital of Shandong University, Jinan, China

**Keywords:** sinus of Valsalva aneurysm, acute myocardial infarction, echocardiography, computed tomography angiography, right coronary artery compression, case report

## Abstract

A sinus of Valsalva aneurysm (SVA) is a rare aortic disease that may be congenital or acquired. Patients with an intact SVA are usually asymptomatic, whereas a ruptured SVA may cause acute chest pain and dyspnea. We present a rare case of acute ST-elevation myocardial infarction and cardiogenic shock in a 51-year-old man. Emergency coronary angiography revealed a giant aneurysm with an absence of flow in the right coronary artery. Both two-dimensional echocardiography and computed tomography angiography showed a giant right SVA, which ruptured into the pericardial sac and led to extrinsic compression of the right coronary artery. Surgical repair combined with coronary bypass grafting was performed. Unfortunately, the patient died from low cardiac output syndrome and postoperative multiple organ failure. This case highlights that the possibility of SVA rupture should be considered in acute myocardial infarction cases and that echocardiography and coronary computed tomography angiography are important in providing an accurate and rapid SVA diagnosis.

## Introduction

A sinus of Valsalva aneurysm (SVA) is a rare cardiac anomaly, with an approximately 0.09% estimated prevalence in the general population (1). Men are two to four times more likely to be affected than women (2, 3). The incidence of ruptured SVA is approximately five times higher in Asians than in Westerners (4). SVAs are most common in the right coronary sinus, followed by the noncoronary and the left coronary sinuses. Although it can be acquired (2, 5, 6) from trauma, infections, and degenerative diseases, it is more frequently congenital. SVA pathology includes the absence of elastic media tissue between the aortic sinus and the hinge line of the aortic annulus, which weakens the aortic wall and results in aneurysmal formation (3). Unruptured SVAs are mostly asymptomatic unless there are complications. In contrast, ruptured SVAs usually cause severe damage and lead to diverse clinical manifestations such as chest pain, dyspnea, tachycardia, or fatigue. Acute myocardial infarction (AMI) may be caused by compression or thrombotic occlusion of the coronary artery (7, 8). An SVA ruptures into the pericardium is fatal owing to pericardial tamponade, with sudden cardiac arrest as the first symptom (9). Herein, we report a giant, ruptured right SVA (RSVA) that extrinsically compressed the right coronary artery (RCA) and presented as acute ST-elevation myocardial infarction and cardiogenic shock.

## Case description

**Figure 1:**
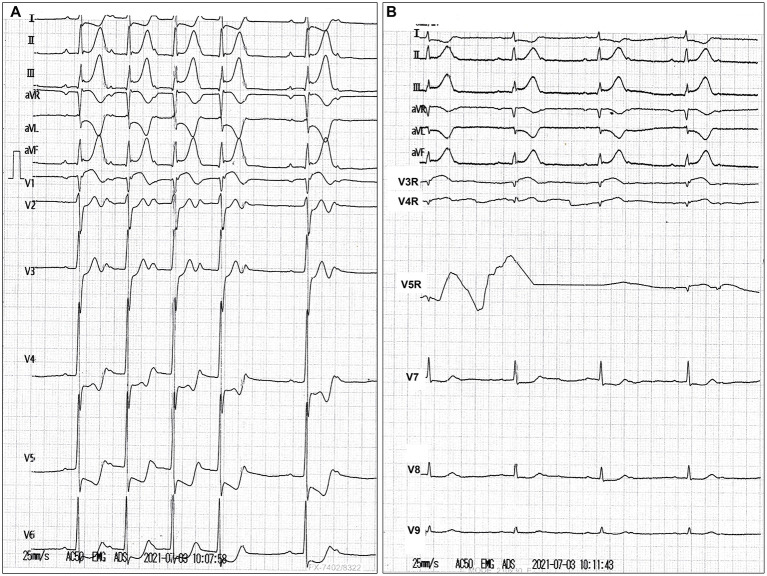
**(A,B)** Electrocardiography images.

A 51-year-old man who had previously been in good condition with no history of smoking or cardiovascular diseases was admitted to a local hospital after presenting with abrupt chest pain, nausea, and emesis for 2 h. He was hemodynamically unstable (blood pressure, 82/50 mmHg; pulse, 48 beats/min). The 18-lead electrocardiogram revealed sinus bradycardia, second-degree atrioventricular block, and ST-segment elevation in leads II, III, aVF, and V3R-V5R (Figure 1). The myoglobin, creatine kinase-MB, troponin I, and N-terminal pro-brain natriuretic peptide levels were 399.6 ng/ml (reference value, <70.0 ng/ml), 66.12 ng/ml (reference value, <5.0 ng/ml), 2.39 ng/ml (reference value, <0.1 ng/ml), and 6,037.0 pg/ml (reference value, ≤300 pg/ml), respectively. The low-density lipoprotein cholesterol level was 2.16 mmol/L (reference value, 0–3.7 mmol/L). The D-dimer level was 0.55 μg/ml (reference value, <0.5 μg/ml). The blood creatinine was 76 μmol/L (reference value, 44–123 μmol/L), with a eGFR of 99.69 ml/(min^*^1.73 m^2^). The patient's liver function was mildly impaired (Child-Pugh A level, no hepatic encephalopathy and ascites, 19.8 μmol/L of bilirubin, 32.3 g/L of albumin, PT-INR 1.04). Loading doses of aspirin and clopidogrel were administered. Emergency coronary angiography revealed persistent swirling of contrast within a large aneurysmal dilatation with an absence of flow in the RCA (Supplementary Video 1). No significant stenosis of the left coronary artery, but a myocardial bridge of the left anterior descending artery was observed (Supplementary Video 2). Bedside echocardiography showed a huge aneurysm (62 mm × 56 mm) close to the right atrium, an enlarged right heart, and severe tricuspid regurgitation. No pericardial effusion was observed. He was subsequently transferred to our hospital.

**Figure 2:**
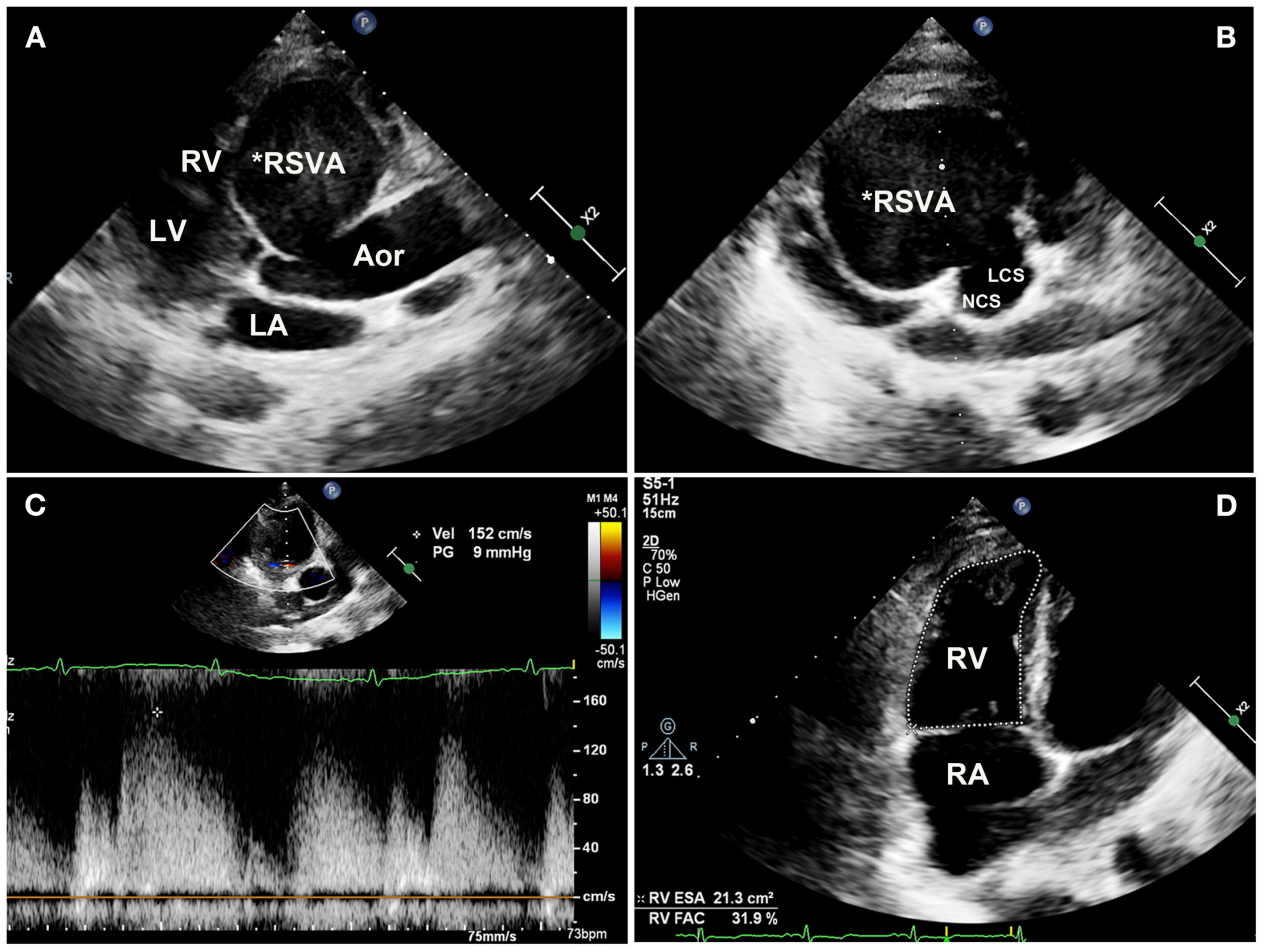
Transthoracic echocardiography images. **(A)** Transthoracic echocardiography showing a giant right sinus of Valsalva aneurysm in the long-axis view and **(B)** the aortic root. **(C)** The compressed RCA is displaced with an accelerating velocity of 150 cm/s. **(D)** The right ventricular systolic fraction is reduced (31.9% RVFAC). RSVA, right sinus of Valsalva aneurysm; RV, right ventricle; LV, left ventricle; LA, left atrium; Aor, aorta; NCS, noncoronary sinus; LCS, left coronary sinus; RA, right atrium; RVFAC, right ventricular fractional area change.

**Figure 3:**
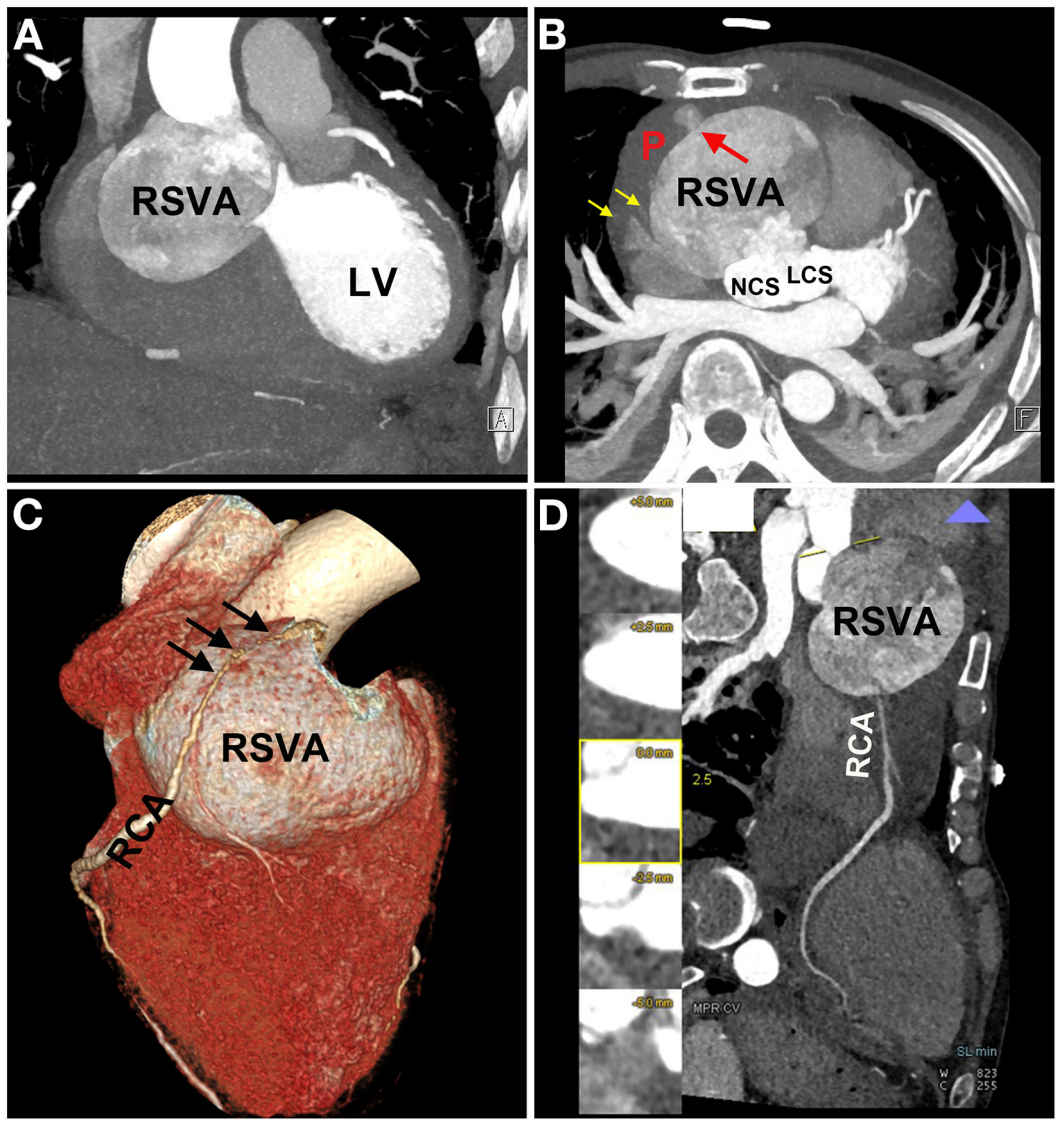
Coronary computed tomography angiography (CTA) images. CTA images showing the RSVA **(A)**, which ruptured to the pericardium [red arrow **(B)**], and some contrast medium leakage into the pericardial sac [yellow arrows **(B)**]; the severely compressed RCA is stretched across the aneurysmal surface [black arrows **(C)**]; the proximal segment of RCA was relatively slender **(D)**. RSVA, right sinus of Valsalva aneurysm; LV, left ventricle; NCS, noncoronary sinus; LCS, left coronary sinus; RCA, right coronary artery.

On physical examination, his blood pressure was 93/70 mmHg with dopamine infusion, and he had a pulse rate of 128 beats/min. No heart murmurs were auscultated. However, the patient's blood pressure became stable after the dopamine infusion was discontinued. Transthoracic echocardiography (Supplementary Video 3) demonstrated a giant RSVA in a prothrombotic state with a small degree of pericardial effusion (Figures 2A,B). The RCA was displaced and compressed with an accelerating velocity (Figure 2C). The inferoposterior left ventricular and lateral right ventricular wall motions were abnormal. The right heart was enlarged and right ventricular systolic function was reduced (right ventricular fractional area change of 31.9%, tricuspid annular plane systolic excursion of 13 mm, Figure 2D, Supplementary Video 4). The left ventricular ejection fraction was 51%. The inferior vena cava diameter was 25 mm, with <50% inspiratory collapse. Coronary computed tomography angiography (CTA) revealed a huge RSVA (65 mm × 70 mm) with contrast medium leakage into the pericardial sac, revealing a rupture of the RSVA (Figures 3A,B); however, it did not show signs of tamponade The RCA was severely compressed and stretched by the giant aneurysm (Figure 3C). and its proximal segment was relatively slender (Figure 3D).

Cardiac surgery was performed using standard cardiopulmonary bypass with moderate hypothermia. Pericardial adhesion was extensive, and there was severe blood loss. After opening the pericardial sac, a huge, dull red aneurysm was seen (Supplementary Figure 1A). Aortic incision was performed, and the aneurysm was explored, revealing no thrombus and a size of 60 mm × 50 mm × 40 mm. The RSVA diagnosis was confirmed, and the RSVA wall appeared fragile and bled profusely. Unfortunately, the perforation was too small to be detected. The orifice of the RSVA neck was ~25 mm in diameter and was closed with a continuous suture and a bovine epicardial patch of suitable size (Supplementary Figures 1B,C). The RCA was confirmed to originate from the aneurysm, and its proximal segment was displaced and severely compressed along with the right ventricle. Thereafter, the RCA was bypassed with a saphenous vein graft. The intraoperative blood flow of the vein graft, measured with a flowmeter, was 130 ml/min. Ventricular fibrillation occurred, and sinus rhythm was restored after one electrical defibrillation. Subsequent cardiopulmonary bypass weaning was successful, and the mediastinal pericardial drainage tube was retained. Exactly 10 units of red blood cells, 1,200 ml fresh plasma, 28 units of cryoprecipitate, and one therapeutic dose of platelets were transfused owing to hemorrhage during surgery. Furthermore, prothrombotic agents, including factor VII, human prothrombotic complex, and human fibrinogen, were also used.

The patient was transferred to the cardiac care unit after surgery. The patient showed signs and symptoms indicative of low cardiac output syndrome (LCOS), manifested as hypotension (80–110/40–70 mmHg with dobutamine), tachycardia (101–123 beats/min in sinus rhythm), poor perfusion (week pulses, cold extremities) and oliguria (0.4 ml/kg/h in the case of diuretics). Central venous pressure values ranged from 16 to 22 mmHg. Laboratory examination demonstrated an increase in lactate of >2 mmol/L on two successive blood gases (2.2–5.0 mmol/L). Bedside echocardiography showed an enlarged right ventricle with poor mobility. The increased right ventricular pressure interfered with the left ventricle volume capacity, despite a 50% ejection fraction. The inferior vena cava diameter was 17 mm with <50% collapse. Several interventions were initiated as the diagnosis of LCOS was established, including administering appropriate dosages of analgesia and sedation, continuing mechanical ventilation, maintaining mild hypothermia in the patient, treating tachyarrhythmias promptly, optimizing preload, intra-aortic balloon pump assistance, and pharmacotherapeutic agents enhancing contractility to improve cardiac output. The patient presented disseminated intravascular coagulation, manifesting reduced platelet count (<100^*^10^9^/L), prolonged thrombin time (>3 s), and elevated fibrin-related markers (d-dimer >10-fold limit of normal). The patient's liver and renal function were aggravated progressively. On the second day, ventricular tachycardia was reported. The patient died on the third day after surgery.

Timeline is shown in Table 1.

**Table 1:** The patient's admission timeline, diagnosis, and treatment.

**Day**	**Events**
30 h prior	The patient presented with abrupt chest pain, nausea, and emesis for 2 h and was admitted to a local hospital Physical examination: blood pressure, 82/50 mmHg; pulse, 48 beats/min Electrocardiogram: sinus bradycardia, second-degree atrioventricular block, and ST-segment elevation in leads II, III, aVF, and V3R-V5R Laboratory tests: elevated myoglobin, creatine kinase MB, troponin I, and N-terminal pro-brain natriuretic peptide Emergency coronary angiography: a giant aneurysm with an absence of flow in the RCA Bedside echocardiography: a huge aneurysm, enlarged right heart, severe tricuspid regurgitation, no pericardial effusion
1	The patient was admitted into our hospital
3	24-h Holter monitor results: sinus rhythm, premature atrial contraction, frequent multifocal premature ventricular contraction, ST-segment, and T-wave changes
8	Transthoracic echocardiography: a giant RSVA in a prothrombotic state, with a small pericardial effusion. The RCA was displaced and compressed, regional wall movement abnormalities, reduced right ventricular systolic function, and dilated inferior vena cava. Tricuspid regurgitation and pulmonary artery hypertension were reduced than before Coronary computed tomography: a huge RSVA (65 mm × 70 mm), which ruptured into the pericardial sac. The RCA was severely compressed and stretched by the giant aneurysm
14	Thoracic computed tomography: bilateral pneumonia, pleural effusion, sachet-like iso-density structure in front of the ascending aorta
18	Preoperative transthoracic echocardiography: a giant RSVA in a prothrombotic state, reduced inferior vena cava's diameter and tricuspid regurgitation
19	Cardiac surgery: The diagnosis of RSVA was confirmed. The perforation was too small to be detected. Combined repair of the ruptured RSVA and coronary artery bypass grafting were performed. Ventricular fibrillation occurred once during the surgery
21	Bedside echocardiography: enlarged right ventricle with poor function. Electrocardiogram: ventricular tachycardia
22	The patient died owing to LCOS and multiple organ failure

## Discussion

Sinus of Valsalva aneurysms leading to AMI are reportedly associated with direct compression (7) or thrombotic occlusion of the coronary artery (8). In our case, the giant RSVA manifested as an acute ST-elevation myocardial infarction with several probable contributing mechanisms. First, RCA compression by the giant aneurysm resulted in a severe coronary oxygen supply-demand mismatch. Second, RCA displacement and stretching across the aneurysmal surface may have disturbed the coronary blood flow. Finally, the echocardiographic prothrombotic state suggested hypercoagulation, which might have led to a detached thrombus forming in the giant RSVA, traveling into the RCA. Another feature was the RSVA rupture into the pericardium. An SVA rupture into the pericardium is fatal owing to pericardial tamponade (9). Cardiogenic shock could have resulted from the AMI or the pericardial tamponade. In this case, despite preoperative transthoracic echocardiography and coronary CTA demonstrating RSVA rupture into the pericardium, the pericardial effusion was small, and the patient's blood pressure gradually stabilized without vasoactive agents or pericardiocentesis. Thus, the leading causes of the cardiogenic shock may have been AMI and severe right heart failure rather than pericardial tamponade. Extensive pericardial adhesion and a tiny perforation might have caused limited pericardial effusion. These two concurrent features make this case uncommon.

Echocardiographic findings of a ruptured SVA were first reported in 1974 (10), and the usefulness of two-dimensional and Doppler echocardiography in assessing such aneurysms was well documented thereafter. Transesophageal echocardiography may provide more information about the aneurysm and its rupture. Both electrocardiogram-gated cardiac CTA and cardiac magnetic resonance imaging can provide excellent anatomic depiction, and cardiac magnetic resonance imaging can provide valuable functional information (11). The echocardiographic exam in this report showed an anatomic abnormality. Therefore, it was possible to evaluate the size and location of the aneurysmal sac. However, myocardial viability detected by single photon emission computed tomography, positron emission tomography, or cardiac magnetic resonance imaging was not performed for financial and safety reasons, which is a limitation of the case. Turbulent fluid was detected inside the SVA. However, the SVA perforation was not detected because of its position or small size. Coronary CTA findings were in line with those of the echocardiography, and the aneurysm was observed to originate from the right coronary sinus, while the aneurysm sac was filled with heterogeneously mixed contrast agents because of its large size. The RCA originated from the right anterior upper part of the RSVA. The proximal segment of the RCA was relatively slender owing to the extrinsic compression of the giant aneurysm, and the relevant positional relationship and SVA perforation location were detected.

The mainstay treatment option for SVA is surgical intervention. Early aggressive treatment is recommended for ruptured SVA to prevent endocarditis or lesion enlargement, which leads to worse symptoms necessitating more extensive repair (2). Surgical strategies include aneurysm resection, patch repair, Bentall procedures, aneurysm closure with concomitant aortic valve replacement, and transcatheter closure (2, 12, 13). So the surgery was finally performed because of the RSVA's rupture and prothrombotic state in this patient. However, the RSVA of this patient might have been through chronic rupture and repaired repeatedly; as seen in the surgery, the pericardial adhesion was extensive, and the wall of the RSVA was thickened, fragile, and abundant in blood vessels. The surgeons thought removing the RSVA at that time was difficult; thus, we performed surgical repair instead. However, the RSVA wall was too fragile to bleed profusely. The limitation of our management was that we focused more on managing AMI and the giant RSVA and neglected the effect of tricuspid regurgitation on right heart function. In hindsight, Bentall procedures and tricuspid valvuloplasty might be more suitable for this patient. The lesson learned from this patient was that we should pay more attention to comprehensive and individualized treatments.

## Conclusion

Acute myocardial infarction caused by a ruptured SVA is a rare cardiac condition that often carries a poor prognosis. This case demonstrates that the possibility of an SVA rupture should be considered in AMI cases. Echocardiography and coronary CTA evaluation are important in providing an accurate and rapid SVA diagnosis. Surgical intervention is still the mainstay and most effective treatment for a ruptured SVA.

## Data availability statement

The original contributions presented in the study are included in the article/Supplementary material, further inquiries can be directed to the corresponding author.

## Ethics statement

The studies involving human participants were reviewed and approved by Ethics Committee of Shandong University Qilu Hospital. The patients/participants provided their written informed consent to participate in this study.

## Author contributions

LM and JY contributed to the manuscript writing. YL and FW contributed to the collection of figures. LM, TL, YW, HS, and CZ contributed to the clinical treatment of this case. YZ contributed to the review of the manuscript. All authors approved the submitted version.

## Funding

This work was supported by a grant from the National Natural Science Foundation of China (81800382), the key Research and Development Plan of Shandong Province (No. 2021SFGC0503).

## Conflict of interest

The authors declare that the research was conducted in the absence of any commercial or financial relationships that could be construed as a potential conflict of interest.

## Publisher's note

All claims expressed in this article are solely those of the authors and do not necessarily represent those of their affiliated organizations, or those of the publisher, the editors and the reviewers. Any product that may be evaluated in this article, or claim that may be made by its manufacturer, is not guaranteed or endorsed by the publisher.

## Abbreviations

SVA: sinus of Valsalva aneurysm
RSVA: right SVA
AMI: acute myocardial infarction
CTA: computed tomography angiography
RCA: right coronary artery
LCOS: low cardiac output syndrome.
