# Supplementary material for: Case report: Acute ST-elevation myocardial infarction and cardiogenic shock caused by a giant right sinus of Valsalva aneurysm and right coronary artery compression
Source: Front Cardiovasc Med. 2022 Oct 18;9:1013044. doi: 10.3389/fcvm.2022.1013044 (PMC9623089; doi:10.3389/fcvm.2022.1013044)
Supplement: Supplementary file 1 [file Data_Sheet_1.DOCX]

Supplementary Material

# Supplementary Figures


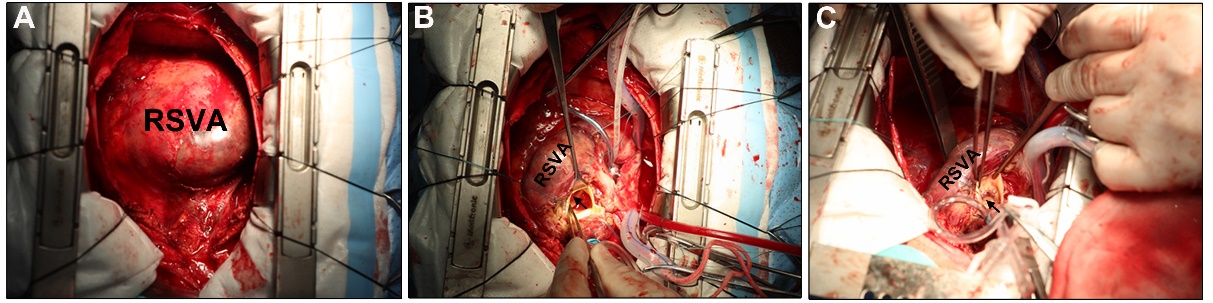


**Supplementary Figure 1.** Surgical images of the RSVA (A), its orifice (black arrow, B) and repaired using a bovine epicardial patch (black arrow, C).

RSVA, right sinus of Valsalva aneurysm

# Supplementary Video legends

**Supplementary Video 1.** Emergency coronary angiography showing a giant aneurysm with an absence of flow in the right coronary artery.

**Supplementary Video 2.** Emergent coronary angiography of the left coronary artery.

**Supplementary Video 3.** Transthoracic echocardiography movie, long-axis view.

**Supplementary Video 4.** Transthoracic echocardiography movie, apical 4-chamber view.
